# Primary brain cell infection by *Toxoplasma gondii* reveals the extent and dynamics of parasite differentiation and its impact on neuron biology

**DOI:** 10.1098/rsob.210053

**Published:** 2021-10-06

**Authors:** Thomas Mouveaux, Emmanuel Roger, Alioune Gueye, Fanny Eysert, Ludovic Huot, Benjamin Grenier-Boley, Jean-Charles Lambert, Mathieu Gissot

**Affiliations:** ^1^ U1019—UMR 9017—CIIL—Center for Infection and Immunity of Lille, University of Lille, CNRS, Inserm, CHU Lille, Institut Pasteur de Lille, F-59000 Lille, France; ^2^ U1167, University of Lille, Inserm, Institut Pasteur de Lille, F-59000 Lille, France

**Keywords:** brain cell, *Toxoplasma gondii*, neuron, differentiation, bradyzoites, parasite

## Abstract

*Toxoplasma gondii* is a eukaryotic parasite that forms latent cysts in the brain of immunocompetent individuals. The latent parasite infection of the immune-privileged central nervous system is linked to most complications. With no drug currently available to eliminate the latent cysts in the brain of infected hosts, the consequences of neurons' long-term infection are unknown. It has long been known that *T. gondii* specifically differentiates into a latent form (bradyzoite) in neurons, but how the infected neuron responds to the infection remains to be elucidated. We have established a new *in vitro* model resulting in the production of mature bradyzoite cysts in brain cells. Using dual, host and parasite RNA-seq, we characterized the dynamics of differentiation of the parasite, revealing the involvement of key pathways in this process. Moreover, we identified how the infected brain cells responded to the parasite infection revealing the drastic changes that take place. We showed that neuronal-specific pathways are strongly affected, with synapse signalling being particularly affected, especially glutamatergic synapse signalling. The establishment of this new *in vitro* model allows investigating both the dynamics of parasite differentiation and the specific response of neurons to long-term infection by this parasite.

## Graphical abstract

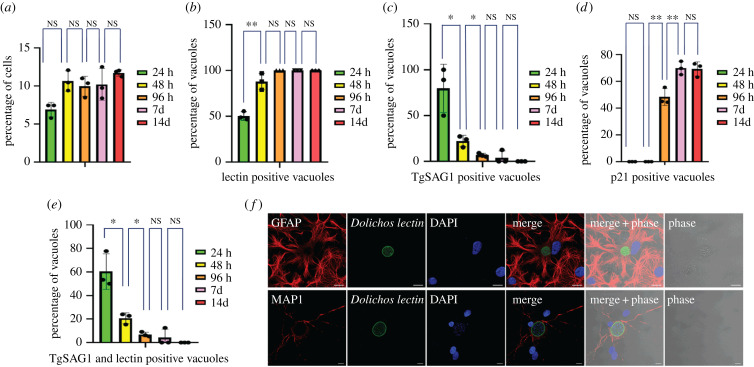

## 1. Introduction

*Toxoplasma gondii* is a unicellular eukaryotic pathogen. It belongs to the Apicomplexa phylum, which encompasses some of the deadliest pathogens of medical and veterinary importance, including *Plasmodium* (the cause of malaria), *Cryptosporidium* (responsible for cryptosporidiosis) and *Eimeria* (causative agent of coccidiosis)*. Toxoplasma gondii* is an obligate intracellular parasite. Although toxoplasmosis is generally asymptomatic, it can lead to the development of focal central nervous system (CNS) infections in immunocompromised hosts. In addition, *Toxoplasma* is also a clinically important opportunistic pathogen that can cause birth defects in the offspring of newly infected mothers. The worldwide seroprevalence of *T. gondii* infection is estimated to be between 30% and 70% in humans, although it differs significantly depending on geographical areas [1].

The life cycle of *T. gondii* is complex, with multiple differentiation steps that are critical to parasite survival in human and feline hosts [2]. Infection by oocysts containing sporozoites shed by cats or by bradyzoites contaminating ingested meat leads to differentiation into rapidly growing tachyzoites that are responsible for clinical manifestations in humans. The conversion of the tachyzoites into bradyzoites, responsible for the acute or the chronic phase of the disease, respectively, is made possible by the unique ability of the tachyzoite to spontaneously differentiate into the bradyzoite form in specific cell types such as muscle cells or neurons. These latent bradyzoites are thought to persist in the infected host for prolonged periods due to their ability to evade the immune system and to resist commonly used drug treatments. Bradyzoites have also the ability to reactivate into virulent tachyzoites and cause encephalitis, in particular in immunocompromised hosts [3]. Therefore, tachyzoite to bradyzoite interconversion is a critical step for the pathogenesis and survival of the parasite. *Toxoplamsna gondii* tachyzoite to bradyzoite stress-induced differentiation has been extensively studied *in vitro* using alkaline stress and other stimuli [4]. However, this process does not produce persisting cysts that express mature bradyzoite markers [5]. It merely reflects the complexity of the process observed *in vivo*. For example, much higher rates of spontaneous differentiation are observed in primary neurons [6]. However, the infection of primary neurons was only performed for short periods (up to 4 days) [7–10]. Therefore, a global understanding of the kinetics and dynamics of differentiation is lacking due to widespread use of the imperfect, but easy to handle, stress-induced differentiation model.

*Toxoplasma gondii* latent infection of the immune-privileged CNS is linked to most complications that can be fatal in the case of reactivation of bradyzoite cysts in immune-deficient hosts. These intracellular parasites migrate to the brain and cross the blood–brain barrier (BBB) by a *Trojan* horse mechanism [11] or by compromising the permeability of the BBB after infection and lysis of epithelial cells [12]. After reaching the CNS, the parasites can invade all nucleated cells, although infection is detected and persist in neurons *in vivo* [13]. Consistent with the ability of this parasite to infect and persist in neurons, *T. gondii* has been linked to behavioural changes in rodent models. The most prevalent study reported the ability of the parasite to specifically manipulate the behaviour of rodents in relation to predator–prey interactions. In these studies, chronically infected mice were specifically impaired for their aversion to feline urine scent [14,15]. Moreover, *T. gondii* infection has been directly implicated in modulating dopamine production [16], decreasing levels of norepinephrine and glutamate [17,18], altering GABAergic signalling [19], thereby inducing an imbalance in neuronal activity [20], inducing neuron apoptosis [21] and altering synaptic protein composition [22]. Chronic toxoplasmosis is also correlated with the establishment of low-grade neuroinflammation characterized by the production of proinflammatory cytokine interferon-gamma (IFN-g). IFN-g is critical to control parasite replication [23] by inducing cell-autonomous immunity of immune resident brain cells notably astrocytes and microglia. Recently, *T. gondii*-induced neuroinflammation has also been linked to behavioural changes in rodents [24,25], indicating that infection probably causes direct and indirect effects on neuronal functions. In humans, a growing number of studies have linked *T. gondii* to psychiatric diseases such as schizophrenia [26,27], behaviour alterations [28], and neurodegenerative diseases such as Parkinson and Alzheimer disease [29], although the causality is not direct and the effect of *T. gondii* infection on human behaviour is likely to be subtle [30]. Indeed, chronic neuroinflammation may also cause neurological disorders by producing neurodegeneration or neurotransmitter abnormalities and therefore altering the neuron functionality [31]. *Toxoplasma gondii* infection may therefore have lifelong effects on the CNS of immunocompetent hosts.

Although global measurement of alteration at the whole-brain level [32,33] clearly indicates broad changes in neuron biological functions, the extent of the modifications of the individual neuron during long-term infection is not understood. Similarly, *in vivo* studies could not address the kinetics of the spontaneous differentiation of the parasite. To address this question, we reasoned that an *in vitro* culture of neurons would require the support of other cells such as astrocytes, which provide metabolic support for neurons and promote the function of synapses [34]. Therefore, we infected a complex primary brain cell culture with *T. gondii* tachyzoites to study the spontaneous differentiation dynamics and the host cell response to infection during differentiation and once the cysts are established. We show here that spontaneous differentiation occurs using this *in vitro* system and can be maintained for at least 14 days. Using RNA-seq, we characterized the dynamic changes in both parasite and host cell gene expression. We investigated the kinetics of parasite differentiation and the alteration of the brain cell gene expression after infection. We showed that this model produced infective bradyzoite cysts after two weeks of culture, mirroring *in vivo* models. Thus, the *in vitro* model we established offers a unique opportunity to dissect the molecular mechanisms of parasite differentiation and the consequences of *T. gondii* infection on neuron biology.

## 2. Material and methods

### 2.1. Parasite strains and culture

*Toxoplasma gondii* tachyzoites of the 76 K strain were propagated *in vitro* in human foreskin fibroblasts (HFF) using Dulbeccos's modified Eagles medium supplemented with 10% fetal calf serum (FCS), 2 mM glutamine and 1% penicillin-streptomycin. Tachyzoites were grown in ventilated tissue culture flasks at 37°C and 5% CO_2_. Prior to infection, intracellular parasites were purified by sequential syringe passage with 17-gauge and 26-gauge needles and filtration through a 3 µm polycarbonate membrane filter (Whatman)

### 2.2. Brain cell culture

Primary neuronal cultures were obtained from the hippocampus of postnatal (P0) rats as described previously [35]. Briefly, after the dissection of the brains, hippocampi were washed three times in HBSS (HBSS, 1-M HEPES, penicillin-streptomycin, and 100 mM sodium pyruvate, Gibco) and were dissociated via trypsin digestion (2.5%, 37°C, Gibco) for 7 min. Next, hippocampi were incubated with DNase (5 mg ml^−1^, Sigma) for 1 min and washed again in MEM medium supplemented with 10% SVF, 1% Glutamax, 0.8% MEM vitamins, 0.5% penicillin-streptomycin and 0.45% d-glucose (Sigma). With a pipette, hippocampi were mechanically dissociated and resuspended in Neurobasal A, a medium supplemented with GlutaMAX and B27 neural supplement with antioxidants (Gibco). Cells were resuspended in culture medium, counted and plated at a density of 100 000 cells cm^−2^ 24-well plates. Plates were pre-coated with 0.1 mg ml^−1^ poly-l-lysine in 0.1 M borate buffer (0.31% boric acid, 0.475% sodium tetraborate, pH = 8.5; Sigma) overnight at 37°C and rinsed thoroughly with water. In total, 200 000 brain cells were seeded per well in 24-well plates. Brain cells were maintained at 37°C in a humidified 5% CO_2_ incubator. Brain cells were grown for 14 days before infection.

### 2.3. Brain cell culture infection

Tachyzoites of the 76 K strain were collected from an infected HFF T25 flask and purified by sequential syringe passage with 17-gauge and 26-gauge needles and filtration through a 3 µm polycarbonate membrane filter (Whatman). Brain cells that were grown and matured in Neurobasal A medium for 14 days were infected by the parasite. For that, the correct amount of tachyzoites was resuspended in 50 µl of Neurobasal A medium and then added onto the brain cell culture. Approximately 2 × 10^5^ brain cells were present in a well of a 24-well plate. Each well was infected by 3 × 10^4^ tachyzoites to a multiplicity of infection of one parasite for around seven cells. The infected culture was maintained at 37°C in a humidified 5% CO_2_ incubator for the duration of the experiment without adding media to avoid disturbing the brain cell culture. A typical experiment yielded 2.5 × 10^4^ cysts per well of a 24-well plate (around 12.5% of the 2 × 10^5^ brain cells).

### 2.4. Mouse infection

Animal housing and experimentation were carried out in accordance with the French Council in Animal Care guidelines for the care and use of animals and following the protocols approved by the Institut Pasteur de Lille's ethical committee (no. 11082-2017072816548341 v2). Brain cells were infected as described above for a duration of 7 or 14 days. Infected and uninfected cells from a single well of a 24-well plate were scraped from the plates and resuspended in 400 µl of sterile PBS. Mice were gavaged with 200 µl of the solution containing the resuspended cells. The content of a single well of a 24-well plate was used to gavage two mice. Uninfected brain cell culture samples were collected at the same time as the 14-day-infected cells. Four weeks after gavage, brains were collected and homogenized individually. Cysts were counted after *Dolichol biflorus* lectin labelling of the cyst wall for 30 min at room temperature to a dilution of 1 : 400 in PBS. One-fifth of the brain of each mouse was scored for the presence of lectin-positive cysts.

### 2.5. RNA sample collection and library preparation

RNA samples were collected after infecting the primary brain cell cultures by the 76 K strain for 24 h, 48 h, 96 h, 7 days and 14 days. Uninfected brain cell culture samples were collected at the same time as the 24 h infected cells time point. Infected and uninfected cells were washed with 1 ml of PBS (two times) and lysed by a direct load of Trizol in the plate. RNA was extracted as per manufacturer instruction and genomic DNA was removed using the RNase-free DNase I Amplification Grade Kit (Sigma). All RNA samples were assessed for quality using an Agilent 2100 Bioanalyzer. RNA samples with an integrity score greater than or equal to 8 were included in the RNA library preparation. Triplicates (biological replicates) were produced for each condition. The TruSeq Stranded mRNA Sample Preparation kit (Illumina) was used to prepare the RNA libraries according to the manufacturer's protocol. Library validation was carried out by using DNA high-sensitivity chips passed on an Agilent 2100 Bioanalyzer. Library quantification was carried out by quantitative PCR (12 K QuantStudio).

### 2.6. RNA-seq and analysis

Clusters were generated on a flow cell within a cBot using the Cluster Generation Kit (Illumina). Libraries were sequenced as 50 bp-reads on a HiSeq 2500 using the sequence by synthesis technique (Illumina). HiSeq control software and real-time analysis component were used for image analysis. Illumina's conversion software (bcl2fastq 2.17) was used for demultiplexing. Datasets were aligned with HiSAT2 v. 2.1.0 [36] against the *T. gondii* ME49 genome from (ToxoDB-39) [37] and against the rat genome (*Rattus norvegicus* Rn6 (UCSC)). Expression for annotated genes was quantified using htseq-count and differential expression was measured by DESeq2. *P*-values for multiple testing were adjusted using the Benjamini–Hochberg method. Differentially expressed genes (DEG) with adjusted *p*-values below 0.05 and log_2_ fold changes (FCs) above 2 were considered in this study. Gene ontology was performed using the PANTHER [38] (Version 15) Overrepresentation Test (Released 20 190 711) surveying GO Slim Biological pathways using the Fisher statistical test for significance. RNA-seq data that support the findings of this study have been deposited in the GEO database under the accession no. GSE168465.

### 2.7. Immunofluorescence analysis

Infected and uninfected brain cell cultures were fixated using 4% PFA for 30 min. The coverslips were incubated with primary antibodies and then secondary antibodies coupled to Alexa-Fluor-488 or Alexa-Fluor-594. Primary antibodies used for IFAs include anti-TgEno2, anti-TgSAG1, anti-MAP2 and anti-GFAP and were used at the following dilutions 1 : 1000, 1 : 1000, 1 : 500 and 1 : 500, respectively. A lectin from *Dolichos biflorus* coupled to fluorescein was also used at 1 : 400 dilution to identify the parasitic vacuoles. Confocal imaging was performed with a ZEISS LSM880 Confocal Microscope. All images were processed using Carl Zeiss ZEN software. Quantification of immunofluorescence assays was carried out manually by counting the concerned signal by visual observation. The signal corresponding to at least 100 vacuoles was counted for each replicate.

### 2.8. Western blot

Total protein extracts representing infected or uninfected cells were resuspended in 1X SDS buffer. The protein samples were then fractionated on a 10% SDS-polyacrylamide electrophoresis gel and then transferred onto a nitrocellulose membrane. The anti-VGLUT1 (cat no. 48-2400, Thermo-Fischer) and anti-GAPDH antibodies were used at a 1 : 1000 dilution. Chemiluminescent detection of bands was carried out by using Super Signal West Femto Maximum Sensitivity Substrate.

## 3. Results

### 3.1. Establishment of the *in vitro* infection model of primary brain cell culture

**Figure 1.**
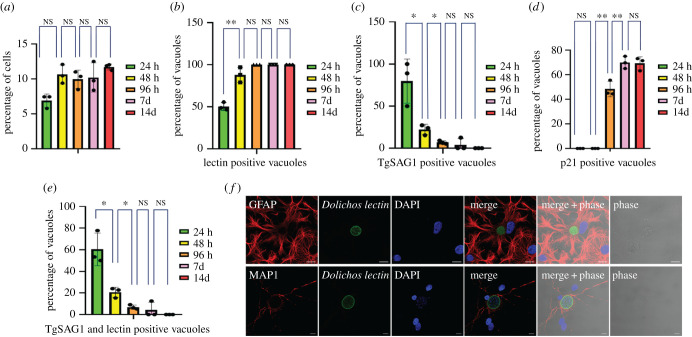
Critical aspects of the primary brain cell culture and its infection by *T. gondii*. (*a*) Graphical representation of the number of infected cells in the brain primary cell culture. Bar graph representing the percentage of infected cells over time after 24 h (green), 48 h (yellow), 96 h (orange), 7 days (pink) and 14 days (red) of infection. A Student's *t*-test was performed; two-tailed *p*-value; NS: *p* > 0,05; mean ± s.d. (*n* = 3, independent experiments). (*b*) Graphical representation of the number of *D. bifluorus* lectin-positive vacuoles. Bar graph representing the percentage of infected cells over time after 24 h (green), 48 h (yellow), 96 h (orange), 7 days (pink) and 14 days (red) of infection. A Student's *t*-test was performed; two-tailed *p*-value; **: *p* < 0,01; NS: *p* > 0,05 ; mean ± s.d. (*n* = 3, independent experiments). (*c*) Graphical representation of the number of vacuoles expressing the tachyzoite marker TgSAG1. Bar graph representing the percentage of TgSAG1 positive parasite vacuoles over time after 24 h (green), 48 h (yellow), 96 h (orange), 7 days (pink), and 14 days (red) of infection. A Student's t-test was performed; two-tailed *p*-value; *: *p* < 0,05; NS: *p* > 0,05 ; mean ± s.d. (*n* = 3, independent experiments). (*d*) Graphical representation of the number of vacuoles expressing the late bradyzoite marker p21. Bar graph representing the percentage of p21 positive parasite vacuoles over time after 24 h (green), 48 h (yellow), 96 h (orange), 7 days (pink) and 14 days (red) of infection. A Student's *t*-test was performed; two-tailed *p*-value; *: *p* < 0,05; **: *p* < 0,01; NS: *p* > 0,05; mean ± s.d. (*n* = 3, independent experiments). (*e*) Graphical representation of the number of vacuoles expressing both the tachyzoite marker TgSAG1 and presenting a lectin labelling. Bar graph representing the percentage of parasite vacuoles double-positive for TgSAG1and *D. bifluorus* lectin labelling over time after 24 h (green), 48 h (yellow), 96 h (orange), 7 days (pink) and 14 days (red) of infection. A Student's *t*-test was performed; two-tailed *p*-value; *: *p* < 0,05; NS: *p* > 0,05; mean ± s.d. (*n* = 3, independent experiments). (*f*) Immunofluorescence labelling of bradyzoite cysts in astrocytes and neurons 7 days post-infection. Confocal imaging demonstrating the presence of bradyzoite cysts (green, labelled with the *D. bifluorus* lectin) in astrocytes (upper panel, red, labelled with GFAP) or neurons (lower panel, red, labelled with MAP2). Anti-GFAP and anti-MAP2 were used as astrocyte and neuron markers, respectively. The scale bar (10 µm) is indicated on the lower right side of each confocal image.

To produce the primary brain cell culture, we extracted brain cells from newborn rats and placed them in culture for 14 days before infection. By immunofluorescence and after quantification, we determined that neurons represented at least 30% of the cells present in culture as identified by the MAP2 marker (electronic supplementary material, figure S1A). Astrocytes, as identified by the GFAP marker, represent more than 50% of the total cells while glial cells and oligodendrocytes represented around 20% of all the cells (electronic supplementary material, figure S1A). This percentage did not vary over time (electronic supplementary material, figure S1A) or after infection (electronic supplementary material, figure S1B). Infection occurred and persisted in neurons and astrocytes and was maintained over time with a similar percentage of cells being infected until the 14 days time point (figure 1*a*). To characterize the *T. gondii* spontaneous differentiation dynamics in this *in vitro* model, we followed the expression of tachyzoite (TgSAG1) and bradyzoite (Cyst wall labelled by *Dolichos bifluorus* lectin and p21, a late bradyzoite marker [39]) markers over time. Spontaneous differentiation occurred within a short time frame in the brain cells with the appearance of parasites expressing a marker of the cyst wall (labelled by the *D. bifluorus* lectin) 24 h after infection representing more than 90% of the parasite population after 96 h (figure 1*b*). Parasites expressing the tachyzoite marker TgSAG1 followed a reverse trend (figure 1*c*). We noted the appearance of the late bradyzoite marker (p21) in cysts 96 h after infection and more than 70% of the cyst population was positive for this marker after 7 days (figure 1*d*). Interestingly, we observed transitioning parasites until 48 h of infection (expressing both tachyzoite and bradyzoite markers TgSAG1 and *D. bifluorus* lectin; figure 1*e*), while all the parasites expressing p21 were also positive for the *D. bifluorus* lectin (electronic supplementary material, figure S1C). Imaging of parasites at 7 days after infection demonstrates that the parasites converted to bradyzoites and established latency in both astrocytes and neurons in this *in vitro* model (figure 1*f*).

### 3.2. Dual RNA-seq on the parasite and host cell during the spontaneous parasite differentiation

**Figure 2.**
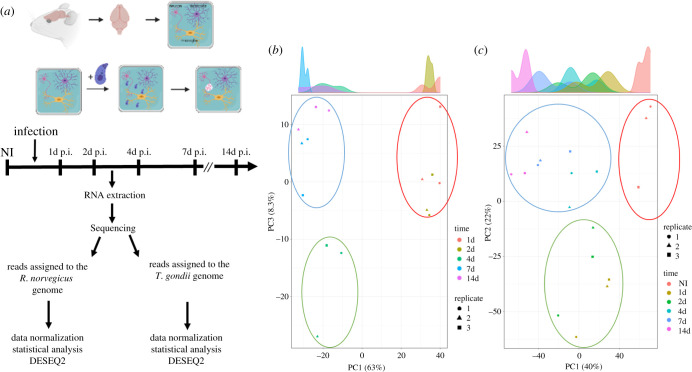
Dual RNA-seq on the uninfected and *T. gondii-*infected primary brain cell culture. (*a*) Schematic of the experiment representing the main steps of the primary brain cell culture and the time points when RNA was extracted. Libraries were created and processed through high-throughput sequencing. Reads were assigned to either the *R. norvegicus* or *T. gondii* genome and DEGs were assigned using DESEQ2. (*b*) PCA of the *T. gondii* triplicate results for each time point. Each replicate is represented by a square, a triangle and a circle. Each time point was assigned a colour: orange (1d), brown(2d), dark green (4d), dark blue (7d) and pink (14d). Based on this analysis, three main groupings were found and represented by a circle: red circle (1d and 2d), green circle (4d) and blue circle (7d and 14d), suggesting sharp transition during differentiation. (*c*) PCA of the *R. norvegicus* triplicate results for each time point. Each replicate is represented by a square, a triangle and a circle. Each time point was assigned a colour: orange (non-infected, NI), brown (1d), green (2d), light blue (4d), dark blue (7d) and pink (14d). Based on this analysis, three main groupings were found and represented by a circle: red circle (non-infected, NI), green circle (1d and 2d) and blue circle (4d, 7d and 14d).

To assess the transcriptome changes during the parasite spontaneous differentiation and the host response to infection, we collected triplicate RNA samples of infected primary CNS cell culture at 1, 2, 4, 7 and 14 days post-infection (figure 2*a*). We analysed transcriptomic profiles of both the parasite and host cells (electronic supplementary material, figure S2A and S2B). Sequencing reads were assigned to the rat or the parasite genome (table 1). For each time point, the infected host transcriptome was compared to a non-infected host cell culture. Reads assigned to the parasite genome were compared to purified tachyzoite-derived sequencing reads. We used a *p*-value cut-off of 0,05 and a minimum twofold change to identify DEG using the DESEQ2 program (table 2; electronic supplementary material, table S1 and S2). We performed a principal component analysis (PCA) to identify how each condition was clustering (figure 2*b,c*). On the parasite side, the PCA analysis revealed that expression was similar between the time points 1d and 2d, while 4d appeared to represent the transition from the tachyzoite to the bradyzoite-specific expression observed at day 7d and 14d (figure 2*b*). On the host side, PCA showed that the response to infection was different for the 1d and 2d time points compared to 7d and 14d (figure 2*c*).

**Table 1.** Number of reads assigned to the *R. Norvegicus* or *T. gondii* genes.

time point (days)	infection	number of reads assigned to the rat genes	% rat	number of reads assigned to the *T. gondii* genes	*% T. gondii*	number total of reads
1	infected	25 252 078	90.73	2 579 227	9.27	27 831 305
1	infected	22 971 803	94.27	1 397 078	5.73	24 368 881
1	infected	25 176 593	96.21	990 451	3.79	26 167 044
2	infected	17 660 544	64.35	9 783 780	35.65	27 444 324
2	infected	19 808 387	79.82	5 007 576	20.18	24 815 963
2	infected	20 621 012	80.37	5 036 121	19.63	25 657 133
4	infected	18 187 041	64.73	10 456 789	35.27	28 643 830
4	infected	17 572 568	74.21	6 106 382	25.79	23 678 950
4	infected	22 141 916	80.89	5 232 408	19.11	27 374 324
7	infected	17 699 964	64.02	9 949 129	35.98	27 649 093
7	infected	16 120 663	68.96	7 257 703	31.04	23 378 366
7	infected	21 899 771	85.98	3 569 992	14.02	25 469 763
14	infected	23 960 501	84.48	4 401 325	15.52	2 836 1826
14	infected	23 324 543	90.36	2 488 854	9.64	25 813 397
14	infected	23 134 477	91.05	2 273 878	8.95	25 408 355
	non-infected	924 580	99.99	3214	0.01	927 794
	non-infected	337 050	99.99	3148	0.01	340 198
	non-infected	160 965	99.99	1398	0.01	162 363
	tachyzoites	1452	0.01	8 825 308	99.99	8 826 760
	tachyzoites	1215	0.01	8 085 704	99.99	8 086 919
	tachyzoites	966	0.01	8 687 601	99.99	8 688 567

**Table 2.** Number of identified DEGs for *R. norvegicus* and *T. gondii*.

comparison	identified genes DESeq2 DEG		up	down	total cut-off >2
*R. norvegicus*					
1d versus NI	12 936	4642	1066	1523	2589
2d versus NI	12 978	3362	1050	1184	2234
4d versus NI	12 960	3114	837	784	1621
7d versus NI	12 985	3995	1150	1232	2382
14d versus NI	13 055	4907	1434	1690	3124
*T. gondii*					
1d versus tachyzoites	7212	2203	610	305	915
2d versus tachyzoites	7381	3241	842	416	1258
4d versus tachyzoites	7745	4634	1220	1005	2225
7d versus tachyzoites	7768	5002	1843	1224	3067
14d versus tachyzoites	7697	4459	1749	1035	2784

### 3.3. Spontaneous parasite differentiation transition is reflected by specific expression patterns

**Figure 3.**
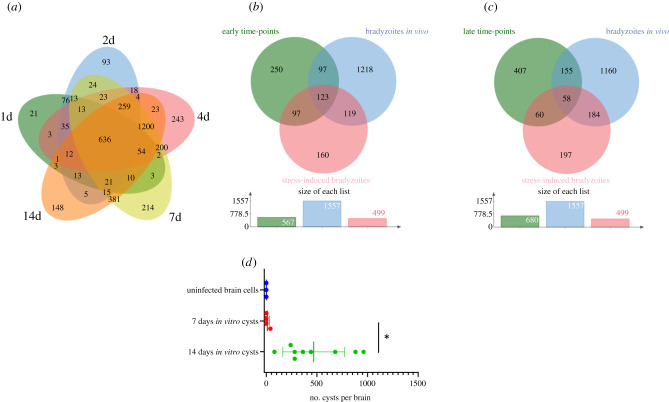
Bradyzoites produced in the infected primary brain cell culture are comparable to *in vitro* and *in vivo* produced bradyzoites. (*a*) Venn diagram of the identified DEGs when comparing tachyzoite to parasite expressed genes at each time point of the brain cell culture. DEGs for the 1d time point are grouped in a green circle. DEGs for the 2d time point are grouped in a blue circle. DEGs for the 4d time point are grouped in a red circle. DEGs for the 7d time point are grouped in a yellow circle. DEGs for the 14d time point are grouped in an orange circle. Several unique or shared DEGs are indicated. (*b*) Venn diagram of the identified upregulated DEGs common for the 1d and 2d time points (green circle), the stress-induced upregulated DEGs (red circle) and the *in vivo-*derived bradyzoites upregulated DEGs (blue circle). The number of unique or shared DEGs is indicated. At the bottom, the size of each list of DEGs is indicated. (*c*) Venn diagram of the identified upregulated DEGs common for the 4d, 7d and 14d time points (green circle), the stress-induced upregulated DEGs (red circle) and the *in vivo-*derived bradyzoites upregulated DEGs (blue circle). The number of unique or shared DEGs is indicated. At the bottom, the size of each list of DEGs is indicated. (*d*) Bradyzoites cysts produced *in vitro* using the primary brain cell culture can transmit the infection after oral gavage. Mice were gavaged by uninfected (blue), 7 days (red) and 14 days (green) infected brain cells. After 6 weeks, mouse brains were collected and the number of cysts per brain was measured. A Student's *t*-test was performed; two-tailed *p*-value; *: *p* < 0,05; mean ± s.d.

We compared the parasite expression profiles obtained for each time point of the brain cell infected culture (figure 3*a*). Differential expression mirrors the timing of spontaneous differentiation. Indeed, most of the changes are initiated at 1d and 2d p.i. and are maintained during later time points (figure 3*a*, 636 DEG). At these time points, parasites are still transitioning (figure 3*a* and table 2; electronic supplementary material, table S1). A turning point is observed at 4d post-infection when the late bradyzoite markers are detected in the *in vitro* culture (figure 1), and parasites further differentiate to mature bradyzoites at days 7 and 14 (figure 3*a*, 1200 DEG common to 4d, 7d and 14d). Little changes are identified in the parasite transcriptome between day 7 and day 14 (figure 3*a*; electronic supplementary material, table S1). The list of common DEGs between each time point encompasses the main bradyzoite markers such as BAG1, ENO1, LDH2 and BRP1 (table 3). By contrast, tachyzoite markers (LDH1, ENO2 and SAG1) were repressed with a different dynamic (table 3). While SAG1 is already repressed 1d after infection, ENO2 and LDH1 were significantly repressed only after 4d of infection (table 3). We performed pathway-enrichment analyses based on the 1200 common DEGs for the 4d, 7d and 14d time points and found that classical pathways known to be repressed such as translation are overrepresented (electronic supplementary material, figure S3). Similarly, the GO-enriched pathways based on the upregulated genes are in line with the carbohydrate metabolism switch known to happen during differentiation (electronic supplementary material, figure S3) [40].

**Table 3.** Gene expression for tachyzoite and bradyzoite markers. Log_2_ FC comparing the expression of *T. gondii* transcripts at each time point of the infected brain cell culture to that of purified tachyzoites. Colour gradient depends on the value of FC. Downregulated values are represented by shades of green. Upregulated values are represented in shades of red. For each transcript, the gene identification number (gene ID) and the corresponding annotation are also presented.

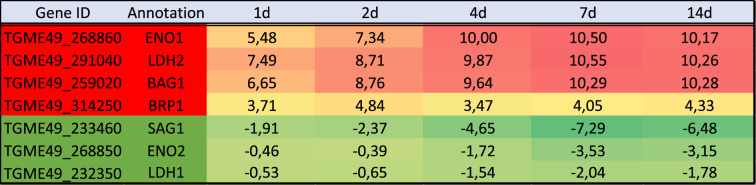

### 3.4. Parasites established in brain cell culture may represent bradyzoites

Expression profiles during stress-induced differentiation were already characterized in numerous studies [41–43]. We compared the expression profiles of up- and downregulated genes after alkaline stress-induced differentiation with the brain cell infected culture RNA-seq results. To account for experimental design and strain differences, we gathered a list of DEGs after alkaline stress-induced differentiation that was common to these three experiments [41–43]. We also compared our dataset to the DEGs that were identified after RNA-seq on *in vivo*-derived bradyzoites [40]. Since there is a clear phenotypic switch between the early time points (1d and 2d) of the infected brain cell culture and the late time points (4d, 7d and 14d), we extracted the DEGs that were common to either early time points (1d and 2d) or late time points (4d, 7d and 14d). This comparison was carried out for upregulated DEGs (figure 3*b*,*c*) and downregulated DEGs (electronic supplementary material, figure S4A and S4B). At early time points, the number of shared upregulated DEGs is equivalent between our dataset and the alkaline stress-induced differentiation or *in vivo-*derived bradyzoites (figure 3*b*). By contrast, at the late time points, the brain cell infected culture DEGs are closer to the *in vivo-*derived bradyzoites DEGs than the alkaline stress-induced differentiation DEGs (figure 3*c*). Similar results were obtained for the downregulated genes (electronic supplementary material, figure S4A and S4B). This indicates that these late time point brain cell-produced bradyzoites may better represent the slow maturation of bradyzoites that is observed *in vivo*. However, brain cell-derived, *in vivo*-derived and stress-induced bradyzoites appear to be three distinct populations with regard to DEGs. Overall, brain cell-derived bradyzoites do not match the *in vivo* bradyzoite profile better than stressed induced-derived bradyzoites.

We investigated if the bradyzoite cysts produced *in vitro* using brain cells could be able to infect mice after oral gavage. In this experiment, the cysts have to go through the digestive system and release the bradyzoites in the gut of the mouse to proceed to the infection of intestine cells. The parasites will then turn into tachyzoites and eventually produce cysts in the brains. We used the cysts formed *in vitro* after 7 or 14 days of differentiation and uninfected brain cells to gavage mice. Six weeks after gavage, we collected the brains of the infected mice and probed for the presence of cysts. All the mice that were gavaged using 14 days *in vitro* cysts were successfully infected and presented cysts in their brain, while only one mouse presented cysts when using 7 days *in vitro* cysts (figure 3*d*) indicating that 14-day cysts may have gone through more maturation steps. No cysts were found in the mice infected by brain cells alone (figure 3*d*).

### 3.5. Expression patterns during parasite differentiation suggest an overhaul of invasion and host cell remodelling activities in the bradyzoite

Tachyzoites have a distinctive ability to modulate the expression of host cells by injecting parasite proteins to hijack the host's regulatory pathways [44]. Very limited information is available about the expression of exported proteins from bradyzoites [45,46] and their abilities to manipulate the host cells. We examined the expression of effector proteins that are known to be exported to the host cell cytosol and nucleus [44]. In our dataset, we found that most of the known effectors were downregulated during differentiation indicating that their expression is no longer needed for bradyzoite development (table 4). Notably, TgIST was the only effector that presented a similar expression level in tachyzoites and bradyzoites and this was for all the time points examined (table 4). As shown before for tachyzoite and bradyzoite markers (table 3), day 4 represented a breaking point where the bradyzoite expression program replaces that of the tachyzoite. Exploring the expression of other potential effectors suggested that a complete transformation in the expression of these proteins is taking place during differentiation (electronic supplementary material, table S3). We also investigated the expression of proteins specialized in the invasion of host cells to verify if the bradyzoites also adapted their invasion machinery. Surprisingly, most of the proteins known to be important for tachyzoite invasion were downregulated (table 5). Instead, a specialized subset of genes (RON2L1, RON2L2, sporoAMA1, AMA2 and AMA4 to a lesser extent) were over-expressed in bradyzoites especially at later time points. These proteins could potentially functionally replace in bradyzoites the tachyzoite specific AMA1 and RON2 proteins (table 5). However, as shown previously in *in vivo*-derived bradyzoites datasets [40], the reads' coverage for sporoAMA1 is only partial in the late time points (14d) indicating that this gene probably produces truncated transcripts and proteins at that stage (electronic supplementary material, figure S5A). By contrast, the AMA2 gene seems to produce full-length transcripts that are preferentially expressed in the late time points of the brain cell infected culture (electronic supplementary material, figure S5B). In line with these profound changes, the expression pattern of ApiAP2 transcription factors that may be responsible for the establishment of the specific expression profile varied also during differentiation (electronic supplementary material, figure S6). ApiAP2 expression profiles grouped in different clusters (electronic supplementary material, figure S6A): a first bradyzoite cluster induced early during differentiation that contained AP2IX-9 [47], a second bradyzoite cluster with factors induced later during differentiation containing AP2XI-4 [48] and a tachyzoite specific cluster with AP2IX-5 [49] and AP2XI-5 and AP2X-5 [50]. PCA based on the ApiAP2 expression profiles mirrored the transition during differentiation (electronic supplementary material, figure S6B). ApiAP2 transcription factors that may control different processes during differentiation may be present in the bradyzoite cluster.

**Table 4.** Gene expression for known tachyzoite effectors. Log_2_ FC comparing the expression of *T. gondii* transcripts at each time point of the infected brain cell culture to that of purified tachyzoites. Colour gradient depends on the value of FC. Downregulated values are represented in shades of green. For each transcript, the gene identification number (gene ID) and the corresponding annotation are also presented.

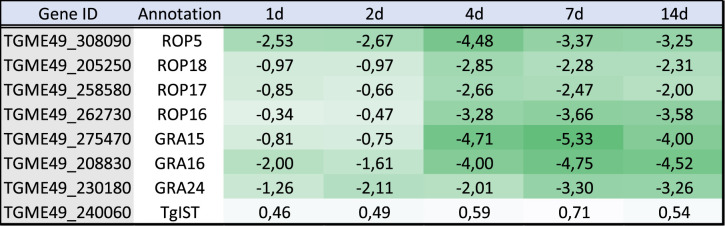

**Table 5.** Gene expression for transcripts encoding proteins known to be involved in invasion. Log_2_ FC comparing the expression *T. gondii* transcripts at each time point of the infected brain cell culture to that of purified tachyzoites. Colour gradient depends on the value of FC. Downregulated values are represented in shades of green. Upregulated values are represented in shades of red. Transcripts that were not detected are indicated by a double dash line (–). For each transcript, the gene identification number (gene ID) and the corresponding annotation are also presented.

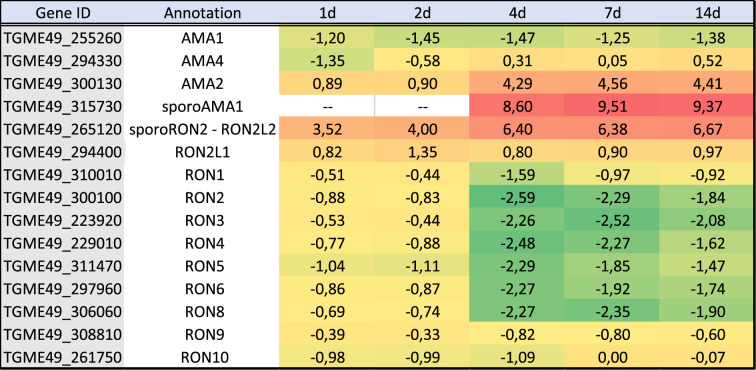

### 3.6. Brain cell culture showed a differential response to tachyzoite and bradyzoite infection

**Figure 4.**
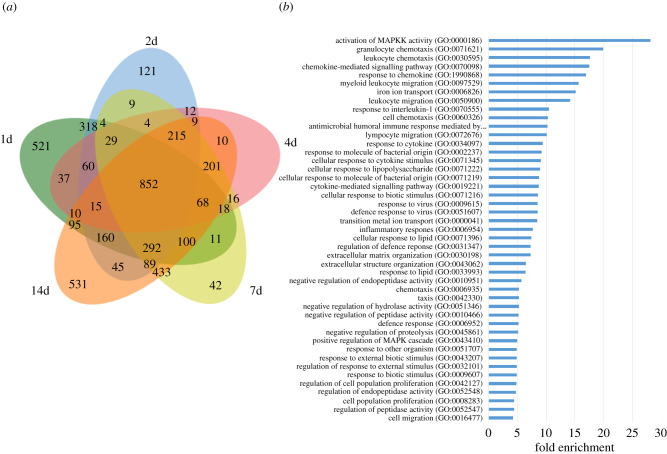
Analysis of identified *R. norvegicus* DEG in the infected primary brain cell culture when compared to uninfected samples. (*a*) Venn diagram of the identified DEGs for each time point. DEGs for the 1d time point are grouped in a green circle. DEGs for the 2d time point are grouped in a blue circle. DEGs for the 4d time point are grouped in a red circle. DEGs for the 7d time point are grouped in a yellow circle. DEGs for the 14d time point are grouped in an orange circle. The number of unique or shared DEGs is indicated. The total number of DEGs for each time point is indicated at the bottom of the figure. (*b*) Enriched GO pathways for upregulated DEGs that are shared for all time point infected brain cells. Pathways were selected with an FDR of 0.05 and a minimum enrichment of 4. The name of each GO pathway is indicated on the left part of the figure. Bars represent the enrichment fold.

On the host side, infection by *T. gondii* tachyzoites triggered a strong response of the host cells (table 2; electronic supplementary material, table S2). This response is mostly stable during the 14d of infection since a large number of DEGs are common between each time point (834 DEGs, figure 4*a*,*b*). However, the early response at 1d (with 521 unique DEGs) and 2d (318 DEGs only present at day 1 and 2 p.i.) may be specific to acute infection (figure 4*a*). We also noted that the later time points (7d and 14d p.i.) presented a unique differential expression pattern (531 DEGs specific from 14d and 433 only common to 7d and 14d). This indicates that a distinctive host response to tachyzoite infection (early time points) is induced when compared to the time when cysts are established (7 and 14 days p.i.). We separated DEGs between upregulated (electronic supplementary material, figure S7A) and downregulated (electronic supplementary material, figure S7B), and we identified similar trends with a number of DEGs being shared between each time point and representing the common response to infection. We also noted that a subset of DEGs was upregulated or downregulated at the first time points while a specific response was also emerging for later time points.

### 3.7. Upregulation of immune-related pathways is a hallmark of *Toxoplasma gondii-*infected brain cell culture

**Figure 5.**
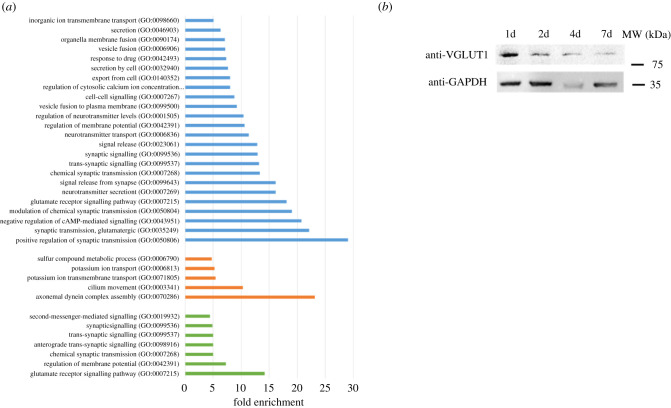
Gene ontology analysis of enriched downregulated pathways in brain cells. (*a*) Enriched GO pathways for downregulated DEGs that are shared for all time point infected brain cells (blue bars), shared for 1d and 2d time points (orange bars) and shared for 7d and 14d time points (green bars). Pathways were selected with an FDR of 0.05 and a minimum enrichment of 4. The name of each GO pathway is indicated on the left part of the figure. Bars represent the enrichment fold. (*b*) Western blot showing the expression of Grm1 (VGLUT1) in neurons after 1, 2, 4 and 7 days of infection. GAPDH is used as a loading control.

We performed a pathway-enrichment analysis on the rat genes that are differentially expressed when comparing the brain cell uninfected cultures to the infected cultures at different time points (figures 4*b* and 5*a*). First, we looked into upregulated genes that were common for all time points and identified that the main response was an immune response to the infection that lasted during the 14 days of infection (figure 4*b*). In particular, the response to chemokine (GO:1 990 868) and the chemokine-mediated signalling pathway (GO:0 070 098) was overrepresented (figure 4*a*). Similarly, upregulated DEGs belonging to the cellular response to cytokine stimulus (GO:0 071 345) and response to cytokine (GO:0 034 097) pathways were also overrepresented. Moreover, the response to interleukin-1 (GO:0 070 555) was also enriched in this dataset. This is in line with the neuroinflammation observed *in vivo* [51] and probably reflects the activation of astrocytes and glial cells present in the culture. This indicates that both microglia and astrocytes present in the brain cell culture responded strongly to the infection *in vitro.* Moreover, a specific response is observed in early time points (days 1 and 2), with a clear enrichment of genes involved in cell cycle and DNA replication arrest (GO:0 045 839 and GO:0 051 985) indicating that infection may induce an arrest of cell division of the brain cells such as glial cells (electronic supplementary material, figure S8A). At later time points, further activation of microglia may take place with the CD80 expression along with Galactin9 expression (electronic supplementary material, figure S8B).

### 3.8. *Toxoplasma gondii* infection induces downregulation of key neuron functions and pathways

Downregulated DEGs common to all time points were analysed using gene ontology. The synapse function was impacted at all time points (figure 5*a*). Notably, the most enriched pathways downregulated were linked to synapse plasticity and transmission (GO:0 050 804, GO:0 007 269 and GO:0 007 268). In particular, the glutamatergic synapse was affected with the downregulation of metabotropic glutamate receptors (Grm1, 2 and 4) and glutamate ionotropic receptor (Grik1, NMDA2C and 2D) as previously described *in vivo* [18]. At later time points, the downregulation of a supplementary metabotropic glutamate receptor (Grm8) together with Homer 1 and 2 protein homologues that link the glutamate receptor to downstream signalling, indicated potential long-term impairment of the glutamate receptor signalling pathway (GO:0 007 215). We inspected the expression of the Grm1 protein during the infection of brain cells and confirmed the downregulation of this protein illustrating the long-term effects of *T. gondii* infection on the glutamatergic synapse (figure 5*b*; electronic supplementary material, figure S8C). Similarly, the glutamate decarboxylase isoforms (Gad1 and Gad2), responsible for GABA production in neurons, were downregulated since 1d recapitulating what was observed *in vivo* [19]. The synaptic signalling was also globally impacted with the downregulation of numerous membrane trafficking regulatory transcripts such as Synaptotagmin-1, Synapsin-2 or Otoferlin.

At early time points (1d and 2d), a specific response to infection consisted of the downregulation of axonemal dynein complex assembly (GO:0 070 286) pathway that suggested an arrest of axonemal assembly. At the same time points, the generation of the action potential and therefore excitability of neurons may be impacted by the downregulation of the potassium ion transmembrane transport (GO:0 071 805) pathway that may occur in neurons or astrocytes. The expression of both the regulatory membrane potential (GO:0 042 391) and chemical synaptic transmission (GO:0 007 268) pathways was also further decreased at late time points of infection, suggesting a strong impact on neuron function.

## 4. Discussion

Tachyzoite to bradyzoite differentiation is a key aspect of *T. gondii* biology and pathogenesis. To date, it has been mainly tackled through the use of an *in vitro* model of stress-induced differentiation that merely reflected the process of spontaneous differentiation observed *in vivo*. Moreover, little is known on the consequences of the long-term infection of targeted host cells *in vivo* (mainly neuron and muscle cells). To better assess the spontaneous differentiation process and the host cell response to infection, we established a complex *in vitro* model where parasites are in contact with multiple cell types normally present in the brain. We reasoned that this complex environment will permit a sustainable long-term infection model. We were able to produce a viable environment promoting neuron survival for a minimum time of 28 days. Using this composite *in vitro* culture system, we successfully established and maintained the infection of neurons and astrocytes by the parasite that progressively express mature bradyzoite markers for at least 14 days. Primary neuronal infection by tachyzoites and bradyzoite differentiation was already experimented in different models for short time frames (up to 4 days) [7–10]. We were able to produce cysts in neurons that could be kept in culture for at least 14 days although longer time frames could be achieved (30 days, data not shown). Strikingly, the cysts produced using this new *in vitro* system have all the molecular features of mature cysts previously observed *in vivo*. They are also infective by oral gavage demonstrating that some of the cysts in the brain cell culture present an intact cyst wall and these *in vitro* produced bradyzoites can readily infect the mouse intestine. Surprisingly, bradyzoites were found in both neurons and astrocytes, a feature that is found in rat, mouse and human primary brain cell culture [6,9,52] but not in mouse brains where bradyzoite survival is only sustained in neurons [13]. Immune cells, that are absent in the primary brain cell culture, may be crucial to eliminate the infected astrocytes *in vivo*.

We showed that parasite expression of bradyzoite markers appeared early in the differentiation process suggesting that the parasites are switching expression patterns at the beginning of the infection process. We observed parasites that were able to co-express markers of both tachyzoite and bradyzoite forms. This illustrates that differentiation is a dynamic process during which tachyzoites expressing bradyzoite markers can be observed until 4 days into the transition. RNA-seq also demonstrated that tachyzoite marker expression is only significantly repressed after 4 days. Such co-expression has also been observed during differentiation *in vivo* [53]. After 7 days, the expression profiles revealed by RNA-seq suggest that the parasites present in the brain cell culture have mainly switch to a bradyzoite-specific expression program. We did not observe major differences in gene expression between 7 and 14 days of culture (electronic supplementary material, table S1). However, only the 14-day bradyzoites containing cysts were competent for mouse infection through gavage, indicating that a maturation process, which is not reflected by transcriptional changes, is still undergoing after 7 days. This post-transcriptional maturation process may involve the modification of the cyst wall.

The parasites produced after 14 days of *in vitro* culture are therefore infectious by oral gavage. In this proof of principle experiment, we showed that using half of a single well of 24-well plate of brain cell-derived bradyzoites is sufficient to produce cysts *in vivo* after oral gavage. However, more work is needed to establish how many brain cell-derived cysts are sufficient to infect a mouse. The *in vitro* culture model described here may be a way to reduce experimental mouse usage. The simplicity to produce the starting material (1 well of a 24-well plate can be used to infect two mice) also offers the possibility to test the infectiousness by oral gavage of multiple parasite mutants. Interestingly, similar results were obtained using a human myotube-based *in vitro* culture model [54], indicating that *in vitro* production of infectious cysts is also possible in other cell types for which a tropism exists *in vivo*.

By examining the expression pattern of transitioning parasites, we observed that the expression of ApiAP2 transcription factors was differentially regulated. Two clusters that appeared early and late during differentiation were identified and may coordinate the dynamic expression profiles observed in the brain cell culture. Interestingly, the over-expression of BFD1, the master switch of differentiation [42], was only observed from 4 days onwards, although its expression might be regulated through a post-transcriptional mechanism. This indicates that multiple layers of regulation may be essential to produce mature bradyzoites.

We have also identified that the expression of the major tachyzoite effectors of host cell manipulation was repressed during differentiation except for TgIST. This suggests that the bradyzoites express a new set of proteins to enable their persistence in neurons. It would be interesting to characterize the proteins that are specifically expressed during differentiation and that have the potential to be exported in the host cell. We also observed the same phenomenon for proteins known to be involved in invasion. Invasion proteins such as AMA1 and RON2, which are key to form a tight connection between the invading parasite and host cell membranes, may be replaced in the bradyzoites by AMA2 or AMA4 and RON2L1 or RON2L2. This modification may be necessary for the bradyzoites to invade specific host cells, such as enterocytes, to complete the life cycle. These new findings are critical for understanding the fundamental changes that occur after differentiation. It suggests that bradyzoites remodel their parasite–host interaction machinery to adapt to a narrower host cell range (intestine enterocyte, neurons and muscle cells) compared to tachyzoites.

Neurons are strongly impacted by *T. gondii* infection. We found that both GABA and glutamate signalling were disrupted in the brain cell culture much like what has been observed *in vivo* in *T. gondii-*infected mouse brains. The glutamate signalling is disrupted from the beginning of the infection with the downregulation of both metabotropic glutamate receptors and glutamate ionotropic receptors. The latter was shown to be repressed in mouse-infected brains [55] and participate in a process proposed to contribute to the establishment of psychiatric disorders such as schizophrenia although the effect of *T. gondii* infection on human behaviour is likely subtle [30]. Thus, this study extends the number of receptors that may be downregulated during infection and further emphasize the impact of infection and inflammation on glutamate signalling.

We also discovered that early on in infection, axonemal growth might be repressed. Development, as well as allowing maintenance of correct cilia structure, is essential for the unique neuron sensory properties, suggesting that neurons may respond to infection by limiting their ability to transfer information. Repression of membrane trafficking regulatory mechanisms was also observed suggesting that the synapse function may be disrupted. This may be aggravated when the parasite established a long-term infection since both membrane potential and chemical synaptic transmission are further disturbed at later time points of the infection. Our data expand and confirm the extent of neuronal function disruption during *T. gondii* infection.

 *Toxoplasma gondii* infection has been linked to a change in behaviour in rodents [14,15]. The strong disruption of glutamate and GABA signalling previously reported [19] is confirmed by our study and may provide a link between the behaviour changes and the infection by *T. gondii*. Since we also observed a signature of a strong neuroinflammation as was shown *in vivo*, it is difficult to define the contribution of the direct infection of neurons and the indirect effects of neuroinflammation on the neuronal pathways. Recent data [24,25] indicate the importance of neuroinflammation in *T. gondii*-induced behavioural changes.

We have established that parasites spontaneously differentiate when infecting a primary brain cell culture. Differentiated parasites present the hallmarks of bradyzoites and persist in culture for prolonged periods. Therefore, this *in vitro* system provides a unique opportunity to dissect the dynamic features of parasite differentiation, but also the direct effect of infection on neuron biology. It could also be of interest for the screening of novel molecules that may be able to eliminate the parasite cyst once it is established in the neurons.

## Data accessibility

RNA-seq data that support the findings of this study have been deposited in the GEO database under the accession no. GSE168465.
